# Naphey’s Modern Therapeutics

**Published:** 1894-02

**Authors:** 


NAPHEYS' MODERN THERAPEUTICS, Medical and Surgical, including the Diseases of Women and Children, A Compendium of Recent Formulæ and Therapeutical Directions from the practice of eminent contemporary physicians, American and foreign; Ninth Edition, Revised and enlarged, Vol. 2, General Surgery, Gynecology and Obstetrics, By Allen J. Smith, M. D., Professor of Pathology, University of Texas, Galveston; Late Assistant Demonstrator of Morbid Anatomy and Pathological Histology and Lecturer on Urinology, University of Pennsylvania; and J. Aubrey Davis, M. D., Assistant Demonstrator of Obstetrics, University of Pennsylvania; Assistant Physician to the Home for Crippled Children, Philadelphia; Philadelphia: P. Blakiston, Son & Co., 1012 Walnut Street, 1893.

Napheys' Therapeutics has been a general favorite for years. Vol. 1, on General Medicine, and Diseases of Children, was noticed in our Review Department some months ago. The volume before us includes the revisions of the latest previous editions of the Surgical Therapeutics and of the Gynecological and Obstetrical Therapeutics, published together, uniformly, with the ninth edition of the Medical volume. It also contains Dermatological Therapeutics; in fact it is a whole "Practice of Medicine'' within itself.

The greater part of the last edition is retained with the modifications required to render it suitable for contemporaneous practice, and a large amount of new material has been added which surgical and gyneological progress has made necessary. The arrangement of matter has been considerably altered, to suit the ideas of the editors, both in the order of chapters and that of individual citations. The latter have been grouped so as to bring therapeutic consideration of similar symptoms, or indications of the diseases, closer together; the arrangement of the chapters was thought to be more convenient for reference. In its new form, and with its many new features and modifications, it will doubtless meet as hearty approval at the hands of the profession as did the previous editions. Vol. 2 has over 1000 pages; it is nicely printed and is strongly and neatly bound, the cloth edition being reinforced with morocco back and corners. Price, cloth, $6.

